# Stage-Specific Dynamic of Gut Microbiota Across the Musk-Secretion Cycle in Forest Musk Deer (*Moschus berezovskii*)

**DOI:** 10.3390/microorganisms14081835

**Published:** 2026-08-19

**Authors:** Gangning Wei, Jianhao Ji, Mengheng Feng, Zhixiang Xiao, Biao Chen, Huirong Mao, Yunlin Zheng, Xiaolong Hu

**Affiliations:** 1 Jiangxi Provincial Key Laboratory of Conservation Biology, Jiangxi Agricultural University, Nanchang 330045, China; wgn_49@163.com; 2 College of Animal Science and Technology, Jiangxi Agricultural University, Nanchang 330045, China; 15522611146@163.com (J.J.); 18203937207@163.com (M.F.); zhixiang_x@163.com (Z.X.); chenbiao@jxau.edu.cn (B.C.); huirongm82@126.com (H.M.); 13907098011@163.com (Y.Z.)

**Keywords:** forest musk deer, musk secretion, gut microbiota, 16S rRNA sequencing, fasting adaptation

## Abstract

Musk secretion in forest musk deer (*Moschus berezovskii*) involves unique physiological processes, yet the paradox of heightened energy expenditure concurrent with voluntary fasting during this period remains poorly understood. Given the role of gut microbiota in host energy metabolism and health, we characterized its compositional and functional dynamics across the musk-secretion cycle. Fecal samples were collected from male forest musk deer at three stages—before (BMS), during (DMS), and after musk secretion (AMS)—and analyzed via full-length 16S rRNA gene sequencing. Results showed that alpha diversity differed significantly among groups (Simpson index: *p* = 0.027). Beta diversity analysis revealed significant community restructuring across stages (Adonis, R^2^ = 0.173, *p* < 0.01), while LEfSe analysis (LDA > 4.0) identified a series of stage-specific taxa, further corroborating the existence of inter-group differences. In terms of species composition, Firmicutes and Bacteroidetes dominated across all stages. Significant inter-stage differences were observed in Verrucomicrobiota (highest in DMS), Patescibacteria (lowest in DMS), Proteobacteria (progressively declining), and Actinobacteria (progressively declining). At the genus level, *Akkermansia*, *Candidatus Saccharimonas*, *Roseburia*, and *Butyricicoccus* showed significant variation across the musk-secretion cycle. Notably, *Akkermansia muciniphila* varied significantly across stages despite high inter-individual variability. Functional prediction revealed the enrichment of hydrocarbon degradation and nitrogen fixation in BMS, and anaerobic respiration and reductive acetogenesis in DMS. This study provides foundational insights into the gut microecological mechanisms underlying musk secretion in forest musk deer.

## 1. Introduction

Musk is a pheromone-like substance secreted by male musk deer, which is valued in traditional Chinese medicine, while it also serves as an indispensable fixative in the global perfume industry [1]. Forest musk deer (*Moschus berezovskii*) are the primary source of natural musk. However, due to historical overhunting and habitat destruction, wild musk deer populations once declined dramatically [2]. Although conservation efforts and the development of captive breeding programs in recent decades have led to a notable recovery of the musk deer population in China, the imbalance between musk supply and demand remains severe. Consequently, improving musk yield in captive musk deer through scientific approaches has become a shared focus for industry and academia [3].

Male forest musk deer undergo a cyclical musk-secretion process between May and July each year. During this period, the musk gland secretes a white viscous musk liquid that is stored in the musk pod for subsequent maturation—this represents a critical phase for musk production [4]. Studies have showed that captive male musk deer typically initiate musk secretion in June, accompanied by a sharp decline in feed intake and a brief period of complete fasting [5]. Paradoxically, musk secretion is a highly energy-demanding physiological process involving extensive cellular synthesis and secretory activities, meaning that the animal reduces or even ceases energy intake precisely when energy expenditure peaks [6,7]. Behavioral observations have revealed altered activity patterns during the musk-secretion period, including diminished crepuscular activity peaks and extended resting periods—clearly representing an energy-conserving strategy [8]. These distinctive manifestations must involve endocrine reorganization and profound changes in energy requirements, rendering the musk-secretion period a unique physiological window. Similar to fasting behaviors induced by hibernation or migration in other animals [9,10], the fasting observed during musk secretion in forest musk deer is not pathological anorexia or a stress response, but rather an active adaptive strategy that is tightly synchronized with the musk-secretion cycle and serves a clear biological function. This type of fasting has been defined in the literature as a “breeding season fast” [11]. Its challenge lies in the complete reliance on endogenous resources to sustain physiological demands. Although different types of fasting phenomena vary in their characteristics, they all require precise physiological regulation to maintain internal homeostasis, particularly with respect to substance and energy metabolism [12,13].

The gut microbiota participates in various biological processes, including energy metabolism, digestion and absorption, and physiological homeostasis. Its composition is influenced by multiple factors such as host diet and health status [14,15]. Evidence suggests that fasting itself can induce changes in gut microbial composition [16,17]. For instance, during hibernation in Daurian ground squirrels (*Spermophilus dauricus*), substantial remodeling of the gut microbiota occurs across distinct intestinal segments. Specific compositional shifts are thought to be associated with decreased intestinal peristalsis and a transition in energy supply strategies during the torpid state, thereby facilitating urea nitrogen recycling and sustaining energy balance under prolonged fasting conditions [18]. Similarly, in the food-storing hibernators Siberian chipmunk (*Tamias sibiricus*), despite periodic feeding during interbout arousals, notable changes in the abundance and composition of certain microbial taxa were observed. These findings suggest that even brief fasting intervals during hibernation are sufficient to drive microbiota remodeling toward a preference for host-derived substrates [19]. In murine models, intermittent fasting has been shown to independently shape the gut microbial through two distinct phases—fasting and re-feeding—generating unique microbial signatures with potential health benefits [20]. Based on the above background, it is reasonable to hypothesize that the different stages of the musk-secretion cycle in forest musk deer are accompanied by similar structured remodeling of the gut microbial community.

In recent years, research on the gut microbiota of forest musk deer has made some progress, covering aspects such as microbial composition, function, health associations, and responses to external factors including environment and diet [21,22,23]. Studies on musk secretion have mainly focused on its molecular regulatory mechanisms, musk composition, and physicochemical properties [1,24,25]. It has been reported that the fungal composition of musk deer feces is associated with individual musk quality and yield [26], and certain specific gut microbial taxa have been proposed as biomarkers for high musk-production efficiency [27]. Beyond these, few studies have linked the gut microbiota to the physiology of musk secretion in forest musk deer. In the present study, we employed full-length 16S amplicon sequencing to analyze fecal samples collected from forest musk deer before, during, and after musk secretion, aiming to elucidate the dynamics of gut microbial composition across the three stages and the physiological adaptation mechanisms of the gut microbiota to musk secretion. The findings are expected to provide practical insights for optimizing feeding management strategies during the musk-secretion period in captive forest musk deer.

## 2. Materials and Methods

### 2.1. Sample Collection

The sampling site of this study was located at a forest musk deer breeding base in Feng County, Baoji City, Shaanxi Province, China. Ten healthy adult male forest musk deer were housed and individually marked with ear tags (S1–S10). Continuous daily sampling was conducted to fully cover the musk-secretion cycle. Forest musk deer were fed a fixed amount of feed at regular times daily. The onset of musk secretion was identified by obvious feed leftovers and loss of appetite, along with a strong musky odor from the enclosure—all readily observable by the keepers. The end of secretion was marked by dissipation of the odor and complete feed consumption. The period from the first appearance of these signs was defined as the during musk secretion stage (DMS). The before musk secretion stage (BMS) refers to the week before onset, and the after musk secretion stage (AMS) refers to the week after termination. To address the issue of insufficient fecal output during DMS due to fasting, we collected all feces excreted throughout the entire DMS period and preferentially selected specimens from the middle portion of this stage to meet the minimum sample requirement for DNA extraction and sequencing. In total, 30 fecal samples were collected from the 10 deer across the three stages, with one sample per individual per stage (n = 10 per group). All musk deer received an identical diet throughout sampling, consisting of roughage (commercially dehydrated elm and mulberry leaves) and concentrated supplements (carrots, pumpkins, feed additives proportionally mixed with crushed grains), with ad libitum access to water. Enclosures were cleaned daily from 04:00 to 06:00 to minimize disturbance to the animals and facilitate the collection of fresh, uncontaminated feces during daylight hours. Fresh fecal samples were immediately placed into sterile cryogenic vials, preserved at −20 °C, transported to the laboratory on dry ice, and subsequently stored at −80 °C. All samples were processed for DNA extraction and sequencing within six weeks after sample collection. All procedures involving animals complied with the institutional guidelines for the ethical treatment of captive wildlife, and non-invasive fecal sampling caused no distress to the animals.

### 2.2. DNA Extraction and 16S rRNA Sequencing

Total genomic DNA was extracted from fecal samples using the QIAamp Fast DNA Stool Mini Kit (Qiagen, Hilden, Germany) according to the manufacturer’s instructions. DNA concentration and purity were assessed using a NanoDrop 2000 (Thermo Fisher Scientific, Waltham, MA, USA) spectrophotometer, and DNA integrity was verified by 1% agarose gel electrophoresis to ensure clear main bands. The full-length bacteria 16S rRNA gene was amplified using primers F (5′-AGAGTTTGATCCTGGCTCAG-3′) and R (5′-GGTTACCTTGTTACGACTTC-3′). PCR amplification was performed in a 25 μL reaction system containing: 12.5 μL of 2× Phanta Max Master Mix (Vazyme, Nanjing, China), 0.5 μL of forward primer (10 μM), 0.5 μL of reverse primer (10 μM), 1 μL of template DNA (50 ng), and ddH2O to a final volume of 25 μL. The thermal cycling program consisted of initial denaturation at 95 °C for 3 min; 35 cycles of denaturation at 95 °C of 30 s, annealing at 55 °C for 30 s, and extension at 72 °C for 90 s; followed by a final extension at 72 °C for 10 min. The amplified products were purified and submitted to Wuhan Benagen Technology Co., Ltd. (Wuhan, China) for subsequent library construction and full-length sequencing on the Nanopore platform.

### 2.3. Bioinformatics Analysis and Statistical Data

Raw data obtained from Nanopore sequencing were subjected to base calling and barcode identification using Dorado (v1.0.0). Adapter and barcode sequences were removed using Porechop (v0.2.3) [28], and sequences with quality scores below 10 were filtered out using NanoFilt (v2.8.0). Primer sequences were identified and trimmed using Cutadapt (v3.5) to obtain high-quality clean reads [29]. For bacteria 16S rRNA gene sequences, alignment against the Silva database (v138) was performed using Lastal (v1648) software [30], and the best-scoring alignment result was used for taxonomic annotation to construct a feature table. Features with an abundance ≤ 1 in any sample were filtered out, and rarefaction was performed based on the minimum sequence count across samples to generate the feature table and abundance data used for downstream analyses.

Taxonomic annotation was performed for each feature, and relative abundance tables were generated and visualized as bar plots. Differential abundance analysis was conducted for the top ten most abundant taxa at the phylum, genus, and species levels. Microbial community diversity was assessed through both alpha and beta diversity metrics. Alpha diversity was evaluated using the Chao1, ACE, Shannon, and Simpson indices, with inter-group differences visualized via boxplots. Beta diversity was analyzed using principal coordinate analysis (PCoA) based on the Bray–Curtis dissimilarity matrix, which was computed using the vegan package (v2.6.4) in R, and the statistical significance of group separation was tested by PERMANOVA (adonis function). LEfSe analysis was performed on the Galaxy platform (v25.0) to identify biomarkers across different musk-secretion stages [31]. Functional prediction was conducted using FAPROTAX (v1.2.1) for ecological functional annotation of the microbial community [32]. Random forest analysis based on the FAPROTAX functional abundance matrix was employed to screen stage-discriminatory functional features.

Given the paired experimental design, the Friedman test was used to assess inter-group differences in the four alpha diversity indices (ACE, Chao1, Shannon, Simpson) and the relative abundance of relevant taxa at each taxonomic level. For indices showing significant differences, pairwise comparisons were performed using the Wilcoxon signed-rank test with Bonferroni correction. The significance level was set at α = 0.05. All visualizations were generated using the R package ggplot2 (v3.4.4) and the OmicStudio online platform (https://www.omicstudio.cn). Statistical analyses were conducted using R software (v4.5.3) and SPSS 27.0.

## 3. Results

### 3.1. Sequencing Data Overview and Microbial Community Diversity

A total of 2,926,137 reads were obtained from 30 fecal samples. Each sample yielded at least 72,608 reads, with an average of 97,537 reads per sample, with an average read length of 1441 bp and an average base quality score greater than 20 (Supplement Table S1). Following alignment and analysis, a feature table comprising 1348 features was generated. A Venn diagram illustrated that 647 features were shared among the three stages (BMS, DMS, and AMS), while 156, 180, and 123 features were unique to the BMS, DMS, and AMS groups, respectively (Supplementary Figure S1). The Q20 values and rarefaction curves for all samples indicated that the sequencing depth was sufficient to adequately characterize the fecal bacterial communities (Supplementary Figure S2).

**Figure 1:**
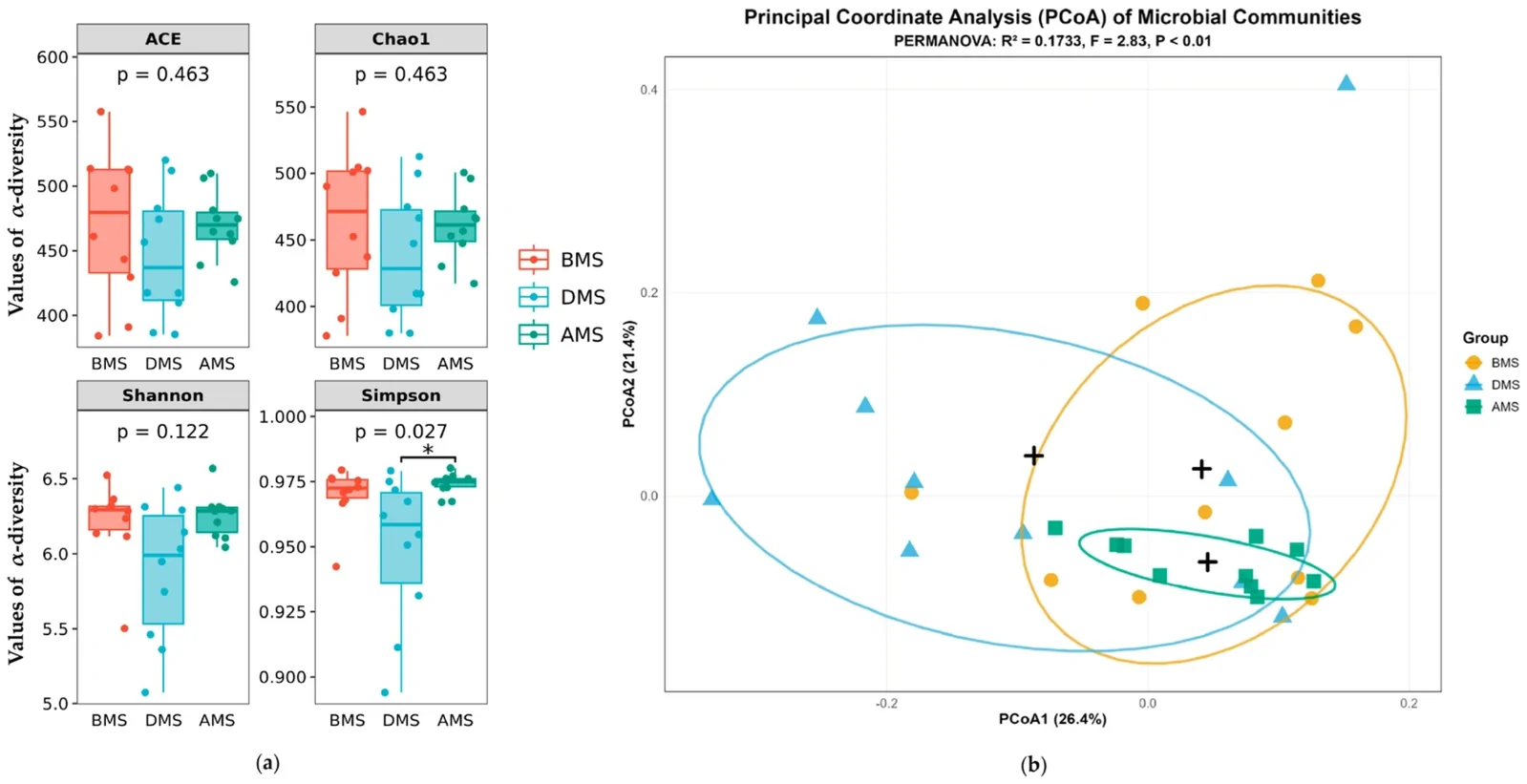
Changes in gut microbiota diversity across the musk secretion cycle. (**a**) Comparison of alpha–diversity indices (ACE, Chao1, Shannon, and Simpson) among the before (BMS), during (DMS), and after (AMS) musk secretion stages. (**b**) Principal coordinate analysis (PCoA) plot based on Bray–Curtis distances, illustrating the beta-diversity of microbial communities. Ellipses represent 95% confidence intervals. PERMANOVA test confirmed a significant effect of the secretion stage on community composition (R^2^ = 0.1733, *p* < 0.01). “*” indicates a significant difference, and “+” denotes the centroid of each group.

Comparison of four alpha diversity indices among the three groups revealed a significant difference in the Simpson index (*p* = 0.027). Specifically, the DMS group exhibited the lowest Simpson index value accompanied by pronounced fluctuations, while the AMS group had the highest Simpson index. No statistically significant differences were observed for the ACE, Chao1, or Shannon indices (Figure 1a). Principal coordinate analysis (PCoA) based on the Bray–Curtis dissimilarity matrix showed partial overlap of microbial communities between groups on the ordination plot; however, permutational multivariate analysis of variance (adonis) confirmed a significant overall divergence in community structure among the three groups (R^2^ = 0.1733, F = 2.83, *p* < 0.01) (Figure 1b). Taken together, these results indicate that the gut microbiota of forest musk deer underwent structural remodeling across different musk-secretion stages.

### 3.2. Microbial Composition and Differentially Abundant Taxa

All features were annotated to 22 phyla, 40 classes, 100 orders, 185 families, 482 genera, and 933 species.

**Figure 2:**
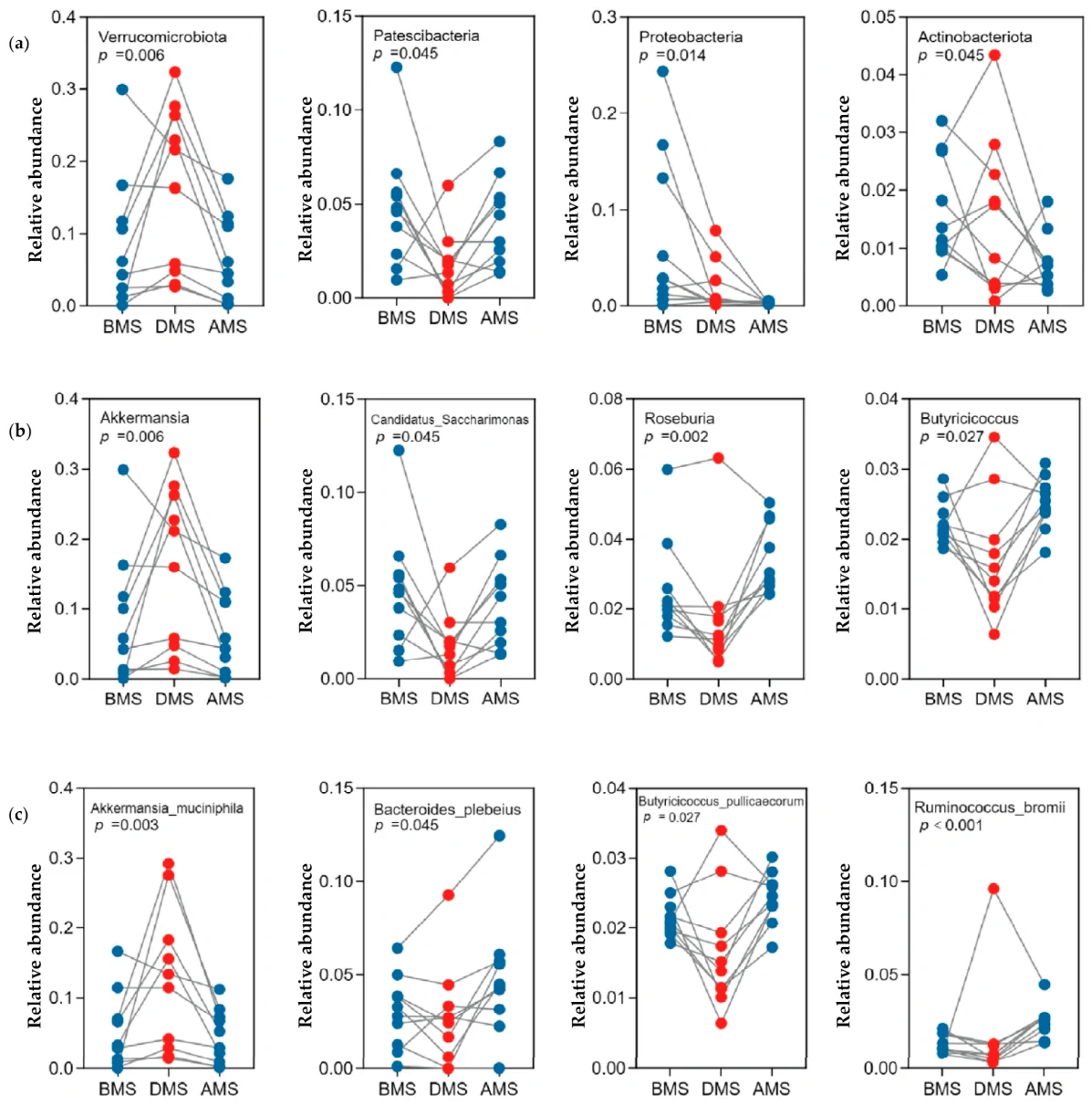
Dynamic trends of key differential taxa across the musk secretion cycle. Connected dot plots (slope charts) illustrate the individual-level changes in relative abundance of the dominant taxa (within the top 10 abundant) that showed significant differences (*p* < 0.05) among secretion stages, at the (**a**) phylum, (**b**) genus, and (**c**) species levels.

Firmicutes and Bacteroidetes were the dominant phyla in the intestinal microbiota of forest musk deer (Table 1). Among the top ten most abundant phyla, significant inter-group differences were observed for Verrucomicrobiota (*p* = 0.006), whose relative abundance reached over 16% during the secretion stage, significantly higher than that in the BMS (8.3%) and AMS (6.8%) stages. Patescibacteria (*p* = 0.045) showed a pattern of decreasing from the BMS (4.8%) to the DMS (1.7%), followed by partial recovery in the AMS (4.0%). Proteobacteria (*p* = 0.014) exhibited a progressive decline from BMS (6.9%) to DMS (1.9%) to AMS (0.3%). Actinobacteriota (*p* = 0.045) and Cyanobacteria (*p* = 0.02) were present at relatively low abundances, making trend interpretation less reliable (Table 1, Figure 2a).

At the genus level, among the top ten most abundant taxa showing significant inter-group differences were *Akkermansia* (*p* = 0.006), *Candidatus Saccharimonas* (*p* = 0.045), *Roseburia* (*p* = 0.002), and *Butyricicoccus* (*p* = 0.027). As the dominant genus within Verrucomicrobiota, *Akkermansia* drove the abundance pattern of this phylum, with both exhibiting highly consistent trends. *Candidatus Saccharimonas* showed a similar pattern to that of Patescibacteria. *Roseburia* abundance decreased from BMS (2.5%) to DMS (1.7%), then recovered and reached a higher level in the AMS (3.4%). *Butyricicoccus* showed a similar trend but with lower overall abundance (BMS: 2.2%; DMS: 1.7%; AMS: 2.5%) (Table 1, Figure 2b).

At the species level, among the top ten most abundant taxa, *Akkermansia muciniphila* (*p* = 0.003) and *Butyricicoccus pullicaecorum* (*p* = 0.027), as the predominant members of the genera *Akkermansia* and *Butyricicoccus*, respectively, exhibited abundance fluctuations highly consistent with their higher taxonomic levels. Additionally, *Bacteroides plebeius* (*p* = 0.045) and *Ruminococcus bromii* (*p* < 0.001) showed significant inter-group differences, primarily driven by contrasts between the DMS and AMS groups (Table 1, Figure 2c).

### 3.3. LEfSe Analysis and Random Forest Feature Selection

**Figure 3:**
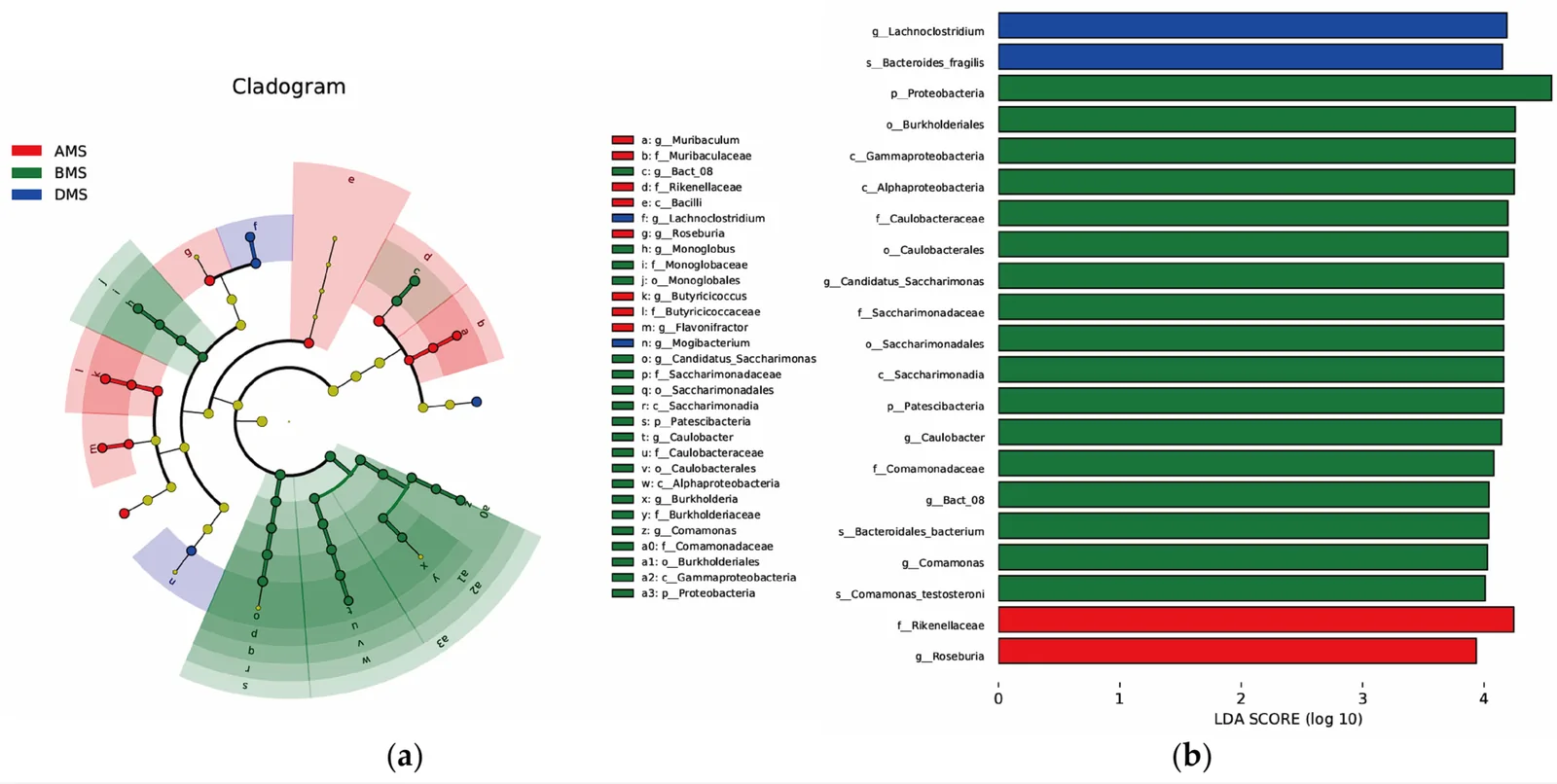
LEfSe analysis of the gut microbiota across different musk secretion stages. (**a**) Cladogram displaying the taxonomic distribution of differentially abundant biomarkers from the Kingdom to genus level among the before (BMS), during (DMS), and after (AMS) musk secretion stages. Circles from inner to outer layers represent different taxonomic ranks (kingdom, phylum, class, order, family, genus), with node size reflecting the relative abundance of the taxon. Different colors indicate the group in which the taxon is significantly enriched (green: BMS; blue: DMS; red: AMS). (**b**) LDA score plot showing the linear discriminant analysis (LDA) effect sizes of taxa with LDA scores greater than 4.0. Bar length represents the contribution of the taxon to the inter-group discrimination, with colors corresponding to the enriched group.

Linear discriminant analysis effect size (LEfSe) was performed to identify biomarkers across groups (LDA > 4.0). The results demonstrated that among the 21 identified biomarkers, several taxa including Patescibacteria, Proteobacteria, *Candidatus Saccharimonas*, and *Roseburia*, concurrently appeared among the significantly differentiated high-abundance species (Figure 3).

**Figure 4:**
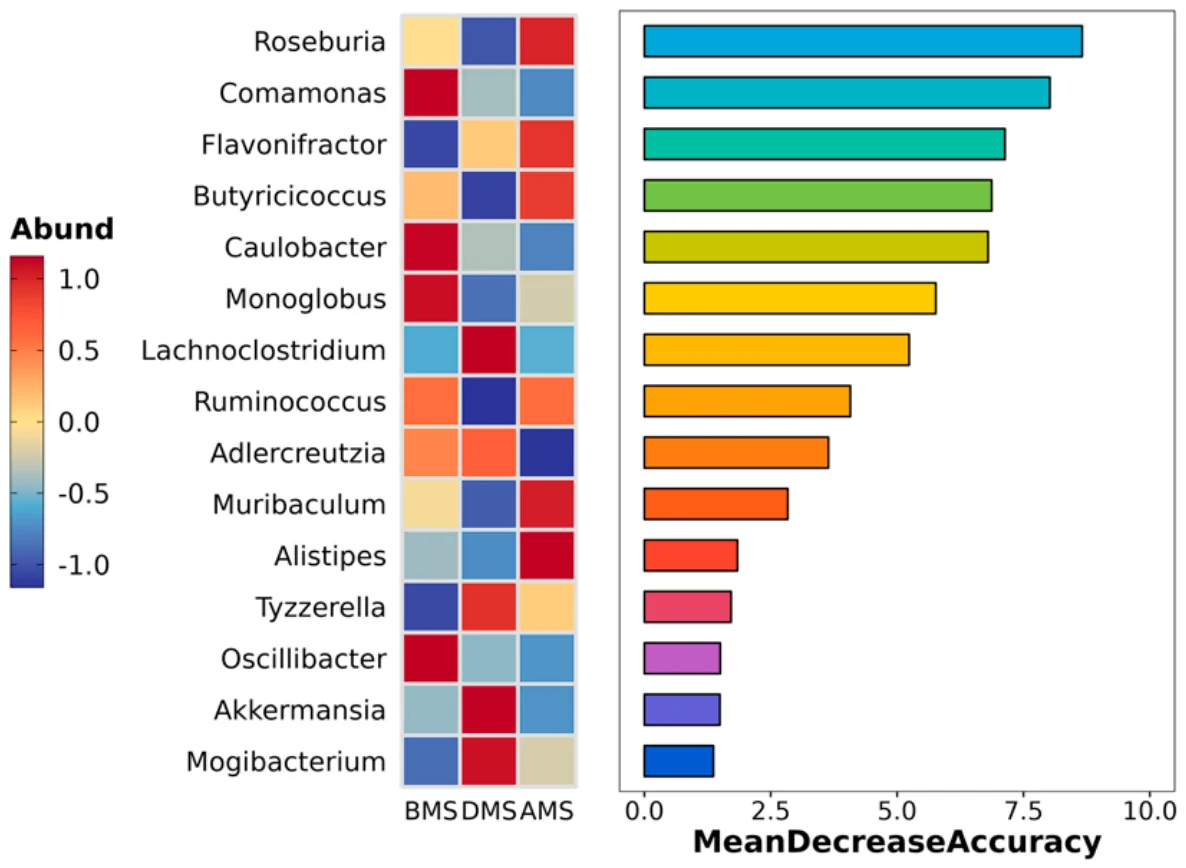
Random forest feature selection at the genus level across different musk secretion stages. Heatmap color represents standardized relative abundance, with red indicating higher abundance and blue indicating lower abundance. Bar length represents MeanDecreaseAccuracy, where longer bars indicate stronger discriminatory power for group classification.

To further identify key microbial taxa associated with musk secretion stages, we performed random forest feature selection at the genus level. Among the results, species including *Roseburia*, *Butyricicoccus* and *Akkermansia* were identified as high−abundance biomarker taxa among the three secretion stages (Figure 4).

### 3.4. Functional Potential Prediction of the Gut Microbiota Based on FAPROTAX

**Figure 5:**
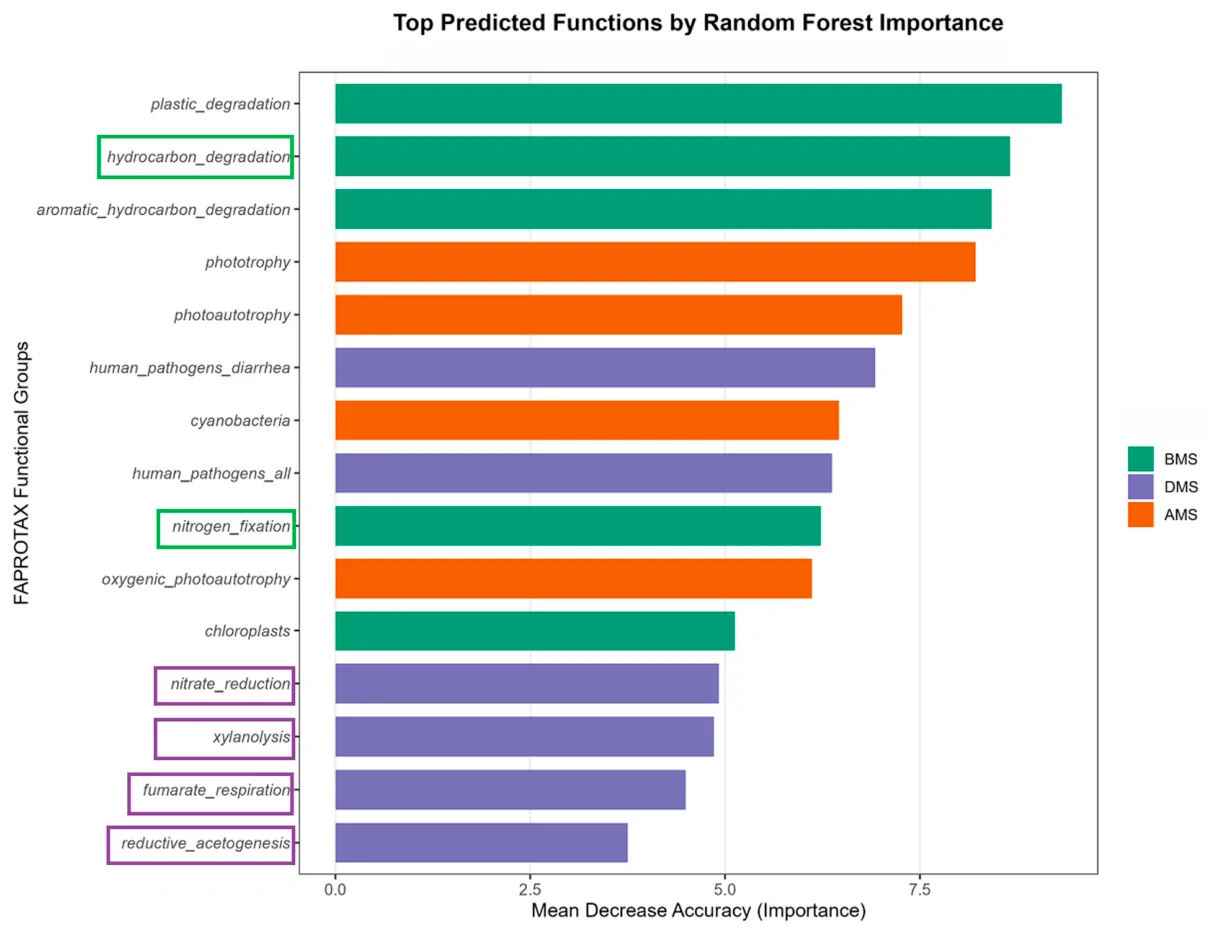
Key predicted FAPROTAX functions identified via random forest analysis. Bar length indicates the importance of each functional group for separating musk secretion stages; color denotes the stage enriched with the corresponding function. Boxed functions highlight the biologically meaningful pathways discussed in the main text.

In the BMS group, the signature functions identified included hydrocarbon degradation, aromatic hydrocarbon degradation, and nitrogen fixation, among others. In the DMS group, the signature functions shifted to nitrate reduction, xylanolysis, fumarate respiration, and reductive acetogenesis. For the AMS group, stage-specific signature functions included phototrophy and others that are not biologically plausible in the mammalian gut. In fact, these likely represent false-positive annotations from the FAPROTAX database resulting from potential contamination sequences derived from plant materials in the diet, and therefore should not be interpreted as indicative of functional activity in vivo (Figure 5).

## 4. Discussion

This study delineated the dynamic changes in the gut microbiota of forest musk deer across three key physiological stages: before, during, and after the musk secretion period. The results revealed that the process of musk secretion was accompanied by a significant remodeling of gut microbial community structure, suggesting that the succession of intestinal microbiota during the musk-secreting cycle is not a stochastic event but rather an adaptive regulation by the host to meet the heightened physiological demands associated with musk production.

In the DMS group, all four alpha diversity indices were reduced and exhibited greater fluctuation. PERMANOVA analysis indicated significant differences in microbial community structure among the three stages, highlighting that the fasting event itself during musk secretion serves as a key driving force for microbial succession. These shifts are analogous to those observed in thirteen-lined ground squirrels, Burmese pythons, and certain frog species, where microbial richness during fasting periods is consistently lower than during non-fasting periods [33,34,35]. Indeed, fasting eliminates dietary-derived nutrient substrates, allowing only a subset of taxa capable of utilizing host endogenous substrates to persist, thereby reducing overall richness [36]. This inference is further corroborated by the absence of a drastic decline in microbial biomass in intermittently feeding hibernators such as Syrian hamsters and Siberian chipmunk, which do not undergo complete fasting [19,37]. The stage-invariant core aligns with the previously described forest musk deer commensal backbone (Firmicutes/Bacteroidetes; *Ruminococcaceae*, *Lachnospiraceae*, *Bacteroides*, *Alistipes*), which plausibly underwrites baseline cellulose degradation, SCFA supply, and intestinal immune homeostasis independent of secretion-stage shift [22,23].

At the genus and species levels, the abundance dynamics of several bacteria taxa with well-established probiotic functions deserve particular attention. Previous studies have reported that the relative abundance of *Akkermansia* is significantly higher during fresh-leaf feeding seasons (summer/autumn) compared to fry-leaf feeding seasons (winter/spring) [22,38], and that geographic differences emerge during ex situ conservation [39]. *Akkermansia* degrades mucin, thereby not only providing energy for itself but also promoting the production of SCFAs such as acetate and butyrate [40]. These SCFAs contribute to enhancing intestinal barrier integrity and repairing epithelial cells, and high *Akkermansia* abundance has been associated with favorable metabolic health, low-grade inflammation, and robust metabolic health [40,41,42]. An increase in the abundance of *Akkermansia muciniphila* was also observed in thirteen-lined ground squirrels during hibernation and fasting [33]. This elevation is thought to be driven by the prolonged lack of dietary polysaccharide substrates due to extended fasting, as well as differences in the gut microbiota’s capacity to utilize host-derived substrates such as mucin and mucus, and support for a similar observation has also been found in a study on Burmese pythons [33,35].

*Butyricicoccus* and *Roseburia*, both well-known butyrate-producing bacteria [43,44], exhibited a marked rebound in abundance during the AMS. Butyrate, as a key SCFA, serves as the preferred energy source for colonic epithelial cells and is essential for maintaining intestinal barrier integrity and function [45,46]. Studies have found that the relative abundance of *Roseburia* in the gut microbiota of wild musk deer is higher than that in captive individuals, and elevated abundance of this genus is generally associated with a healthy gut microbial ecosystem [47]. Therefore, the robust resurgence of these butyrate-producing bacteria in the AMS may position them as “restorers” and “stabilizers”, aiding the host in recovering intestinal homeostasis and energy balance following the energetically demanding musk-secretion activity. *Rikenellaceae*, identified as another biomarker of the BMS group, exhibited abundance correlations with season, age, and sex. This family belongs to the phylum Bacteroidetes and possesses the capacity to degrade complex polysaccharides and proteins, promote intestinal immune development, and participate in lipid metabolism [48,49]. *Lachnoclostridium*, identified as a biomarker for the DMS, has been shown to be significantly more abundant in healthy forest musk deer individuals compared to those with diarrhea [50]. This genus is believed to alleviate hepatic inflammation, improve intestinal barrier function, and inhibit lipid accumulation through butyrate production [51].

The association between fasting and the gut microbiota has emerged as a burgeoning interdisciplinary field [52]. The core mechanism underlying this relationship is that when food supply is interrupted, the nutritional sources available to the microbial community undergo dramatic changes, leading to compositional and functional remodeling—a process that is not merely a passive decline but rather an active adaptive realignment [53,54]. For forest musk deer, the stage-specific remodeling of the gut microbiota observed in this study likely reflects a coordinated host–microbiome response to the unique energy crisis imposed by fasting during the musk-secretion period. Functional prediction using FAPROTAX further corroborated these observations. In the BMS group, functions such as hydrocarbon degradation and nitrogen fixation were identified, suggesting that the gut microbiota actively participates in the decomposition of plant secondary metabolites. In the DMS group, the characteristic functions shifted toward anaerobic respiration and acetogenesis-related pathways. This indicates that under fasting stress during the musk secretion period, the host’s energy supply partially relies on microbial anaerobic fermentation derived acetate, with the gut microbiota contributing to host energy homeostasis through anaerobic metabolic pathways.

Nevertheless, these functional predictions should be interpreted with caution, as FAPROTAX predictions are based solely on 16S rRNA gene data and require confirmation by direct functional assays such as metatranscriptomics or metabolomics. Additionally, given the correlational nature of our study design and the lack of matched host metabolic phenotype data (e.g., body weight, serum energy markers, fecal SCFAs), the observed microbial shifts cannot be exclusively attributed to fasting, so future controlled feeding experiments incorporating physiological and metabolic measurements are needed to validate these findings.

## 5. Conclusions

Our study demonstrates that the gut microbiota of forest musk deer exhibits remarkable plasticity across the unique physiological cycle of musk secretion. The regulated changes in mucosa-associated bacteria represented by *Akkermansia* and butyrate-producing bacteria represented by *Butyricicoccus* and *Roseburia* may represent key mechanisms through which the microbiota assists the host in maintaining intestinal homeostasis and providing energetic support during musk secretion. This study lays a foundation for understanding the microecological regulatory mechanisms underlying this complex physiological process. Such efforts would provide a theoretical basis for developing nutritional intervention strategies—such as dietary fiber modulation or microbiota-oriented supplements—aimed at improving health and musk yield in captive musk deer.

## Figures and Tables

**Table 1:** Relative abundances (mean ± SE) of bacterial taxa at the phylum, genus, and species levels. “ns” indicates no significant difference.

Taxa	BMS	DMS	AMS	Significance
Phylum				
Firmicutes	0.517 ± 0.019	0.539 ± 0.031	0.550 ± 0.018	ns
Bacteroidetes	0.162 ± 0.021	0.150 ± 0.028	0.208 ± 0.008	ns
Verrucomicrobiota	0.083 ± 0.030 a	0.164 ± 0.036 b	0.068 ± 0.019 a	*p* = 0.006
Planctomycetota	0.084 ± 0.018	0.054 ± 0.016	0.092 ± 0.013	ns
Patescibacteria	0.048 ± 0.010 a	0.017 ± 0.006 b	0.040 ± 0.007 ab	*p* = 0.045
Proteobacteria	0.069 ± 0.026 a	0.019 ± 0.008 ab	0.003 ± 0.000 b	*p* = 0.014
Actinobacteriota	0.016 ± 0.003 a	0.015 ± 0.004 ab	0.008 ± 0.002 b	*p* = 0.045
Euryarchaeota	0.003 ± 0.001	0.012 ± 0.006	0.015 ± 0.005	ns
Cyanobacteria	0.003 ± 0.001 ab	0.001 ± 0.000 a	0.005 ± 0.001 b	*p* = 0.02
Genus				
*Akkermansia*	0.081 ± 0.030 ab	0.161 ± 0.037 b	0.067 ± 0.019 a	*p* = 0.006
*Bacteroides*	0.070 ± 0.011	0.094 ± 0.027	0.099 ± 0.012	ns
*Intestinimonas*	0.045 ± 0.003	0.042 ± 0.005	0.052 ± 0.003	ns
*Ruminococcus*	0.046 ± 0.003	0.037 ± 0.012	0.046 ± 0.004	ns
*Sporobacter*	0.035 ± 0.002	0.036 ± 0.005	0.037 ± 0.002	ns
*Candidatus_Saccharimonas*	0.048 ± 0.010 a	0.017 ± 0.006 b	0.040 ± 0.007 ab	*p* = 0.045
*Bact-08*	0.042 ± 0.006	0.021 ± 0.005	0.041 ± 0.006	ns
*Pirellula*	0.026 ± 0.008	0.024 ± 0.009	0.032 ± 0.008	ns
*Roseburia*	0.025 ± 0.004 ab	0.017 ± 0.005 b	0.034 ± 0.003 a	*p* = 0.002
*Butyricicoccus*	0.022 ± 0.001 ab	0.017 ± 0.003 b	0.025 ± 0.001 a	*p* = 0.027
Species				
*Akkermansia_muciniphila*	0.050 ± 0.017 a	0.157 ± 0.039 b	0.045 ± 0.012 a	*p* = 0.003
*Sporobacter_termitidis*	0.035 ± 0.002	0.036 ± 0.005	0.036 ± 0.002	ns
*Bacteroides_plebeius*	0.030 ± 0.006 ab	0.027 ± 0.009 a	0.048 ± 0.010 b	*p* = 0.045
*Bacteroidales_bacterium*	0.041 ± 0.006	0.021 ± 0.005	0.041 ± 0.006	ns
*Akkermansia_glycaniphila*	0.032 ± 0.012	0.032 ± 0.011	0.022 ± 0.007	ns
*Pirellula_staleyi*	0.026 ± 0.008	0.024 ± 0.009	0.032 ± 0.008	ns
*Intestinimonas_butyriciproducens*	0.023 ± 0.002	0.025 ± 0.003	0.032 ± 0.002	ns
*Butyricicoccus_pullicaecorum*	0.022 ± 0.001 ab	0.017 ± 0.003 a	0.024 ± 0.001 b	*p* = 0.027
*Christensenella_minuta*	0.020 ± 0.003	0.023 ± 0.003	0.017 ± 0.001	ns
*Ruminococcus_bromii*	0.013 ± 0.002 ab	0.017 ± 0.009 a	0.025 ± 0.003 b	*p* < 0.001

Statistical analysis was performed using the Friedman test due to the paired nature of the experimental design. Post hoc pairwise comparisons were conducted using the Wilcoxon signed-rank test with Bonferroni correction. Different lowercase letters (a, b) denote significant differences (*p* < 0.05) between stages. Groups sharing the same letter are not significantly different.

## Data Availability

The raw sequence data reported in this paper have been deposited in the Genome Sequence Archive (Genomics, Proteomics & Bioinformatics 2025) at the National Genomics Data Center (Nucleic Acids Res 2025), China National Center for Bioinformation/Beijing Institute of Genomics, Chinese Academy of Sciences (GSA: CRA046307), which are publicly accessible at https://ngdc.cncb.ac.cn/gsa on 15 July 2026.

## Supplementary Materials

The following supporting information can be downloaded at: https://www.mdpi.com/article/10.3390/microorganisms14081835/s1, Table S1: Quality control summary of raw sequencing data derived from forest musk deer fecal samples; Figure S1: Venn diagram of shared and unique features among AMS, BMS, and DMS groups. AMS (After Musk Secretion), BMS (Before Musk Secretion), and DMS (During Musk Secretion). Numbers in overlapping regions indicate counts of features shared between groups. The central region (647) represents core features common to all three stages. Non-overlapping regions denote group-specific features (123 in AMS only, 156 in BMS only, and 180 in DMS only); Figure S2: Rarefaction curves of feature richness across all samples. The x-axis denotes the subsample sequences number, and the y-axis denotes the feature number. Each line represents a single sample.
