# Supplementary material for: Stage-Specific Dynamic of Gut Microbiota Across the Musk-Secretion Cycle in Forest Musk Deer (Moschus berezovskii)
Source: Microorganisms. 2026 Aug 19;14(8):1835. doi: 10.3390/microorganisms14081835 (PMC13515588; doi:10.3390/microorganisms14081835)
Supplement: Supplementary file 1 [file microorganisms-14-01835-s001.zip › microorganisms-4488500-supplementary.pdf]

Table S1. Quality control summary of raw sequencing data derived from forest musk deer fecal samples.

Sample	Raw Reads	Clean Reads	Mean_len(bp)	Mean_Q
S1B	94114	71670	1440	20.55
S2B	100102	75921	1445	20.45
S3B	93691	71253	1443	20.49
S4B	72608	54623	1442	20.42
S5B	82647	60645	1446	20.43
S6B	90945	67857	1436	20.59
S7B	100300	76769	1443	20.58
S8B	100291	76015	1437	20.55
S9B	99679	76696	1442	20.38
S10B	94124	71081	1441	20.46
S1D	100161	76214	1439	20.48
S2D	100054	76743	1445	20.53
S3D	99783	78145	1442	20.45
S4D	99552	77210	1440	20.54
S5D	99509	75681	1444	20.41
S6D	99970	77212	1438	20.46
S7D	99666	77856	1438	20.56
S8D	100299	77793	1436	20.56
S9D	100214	78040	1437	20.56
S10D	99626	76740	1440	20.37
S1A	99747	76812	1444	20.43
S2A	100309	76074	1445	20.41
S3A	99780	76352	1444	20.4
S4A	100129	77250	1445	20.31
S5A	99637	78027	1444	20.44
S6A	99498	76842	1440	20.39
S7A	100260	76486	1443	20.38
S8A	99647	76613	1442	20.41
S9A	99925	76461	1441	20.46
S10A	99870	75606	1442	20.43

Data are presented as the number of reads. Clean reads were obtained after removing low-quality reads, adapter sequences, and reads containing N bases. Mean_len (bp) represents the average length of clean reads (base pairs), and Mean_Q represents the average Phred quality score (Q-value) of clean reads, reflecting the accuracy of base calling. All samples showed high-quality sequencing data suitable for downstream analysis.

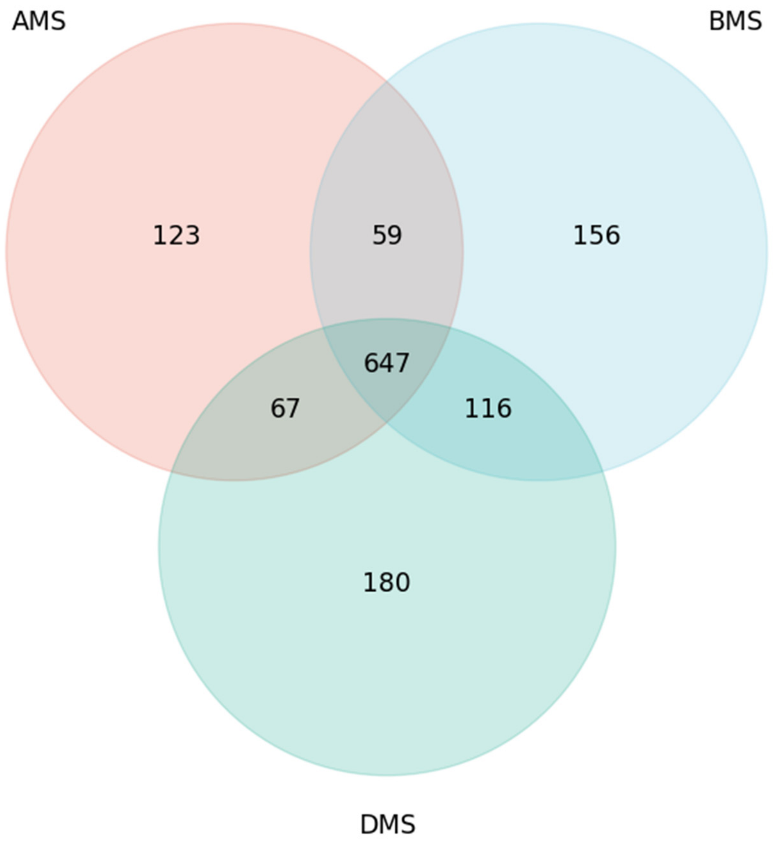

Figure S1. Venn diagram of shared and unique features among AMS, BMS, and DMS groups. AMS (After Musk Secretion), BMS (Before Musk Secretion), and DMS (During Musk Secretion). Numbers in overlapping regions indicate counts of features shared between groups. The central region (647) represents core features common to all three stages. Non-overlapping regions denote group-specific features (123 in AMS only, 156 in BMS only, and 180 in DMS only).

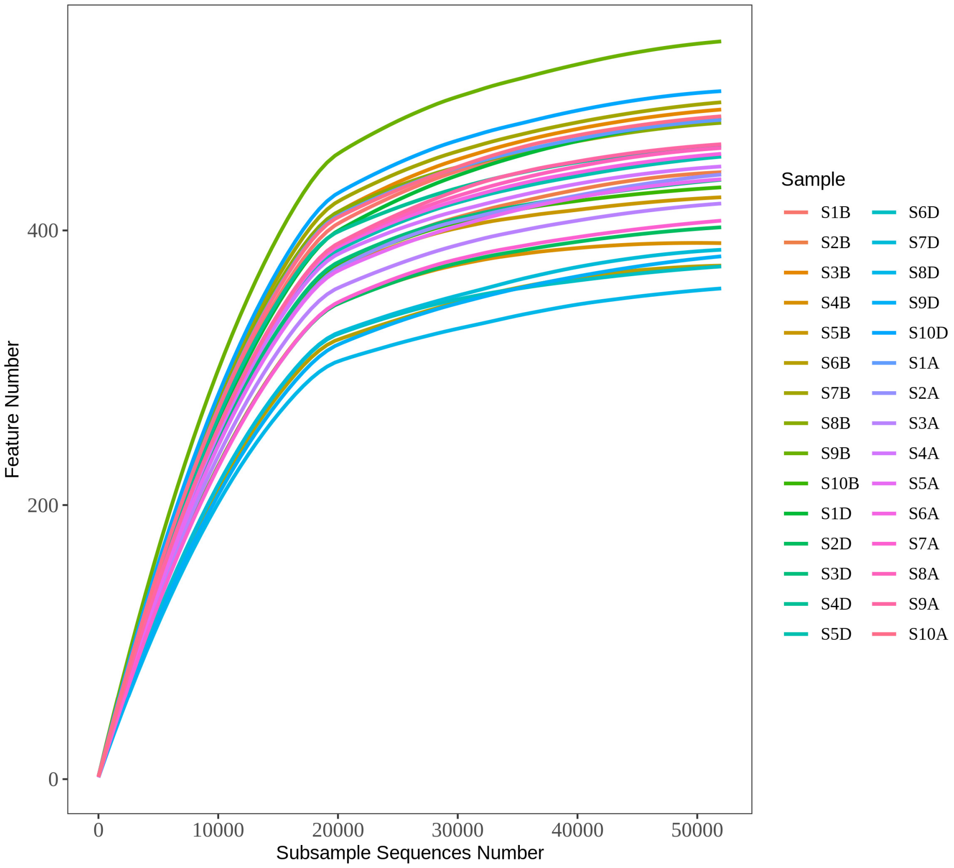

Figure S2. Rarefaction curves of feature richness across all samples. The x-axis denotes the subsample sequences number, and the y-axis denotes the feature number. Each line represents a single sample.
